# A Metaanalysis of Perceptual Organization in Schizophrenia, Schizotypy, and Other High-Risk Groups Based on Variants of the Embedded Figures Task

**DOI:** 10.3389/fpsyg.2016.00237

**Published:** 2016-02-23

**Authors:** Kirsten R. Panton, David R. Badcock, Johanna C. Badcock

**Affiliations:** ^1^Human Vision Laboratory, School of Psychology, University of Western AustraliaPerth, WA, Australia; ^2^Centre for Clinical Research in Neuropsychiatry, School of Psychiatry and Clinical Neurosciences, University of Western AustraliaPerth, WA, Australia; ^3^Cooperative Research Centre - Mental HealthMelbourne, VIC, Australia

**Keywords:** schizophrenia, schizotypy, perceptual organization, global processing, visual integration

## Abstract

Current research on perceptual organization in schizophrenia frequently employs shapes with regularly sampled contours (fragmented stimuli), in noise fields composed of similar elements, to elicit visual abnormalities. However, perceptual organization is multi-factorial and, in earlier studies, continuous contours have also been employed in tasks assessing the ability to extract shapes from a background. We conducted a systematic review and meta-analysis of studies using closed-contour stimuli, including the Embedded Figures Test (EFT) and related tasks, both in people with schizophrenia and in healthy schizotypes and relatives, considered at increased risk for psychosis. Eleven studies met the selection criteria for inclusion in the meta-analysis, including six that used a between-groups study design (i.e., perceptual organization abilities of schizophrenia/high-risk groups were compared to healthy or clinical controls), and five that treated schizophrenia symptoms or schizotypy traits and indices of perceptual organization as continuous variables. Effect sizes and heterogeneity statistics were calculated, and the risk of publication bias was explored. A significant, moderate effect for EFT performance was found with studies that compared performance of schizophrenia/high-risk groups to a healthy or patient comparison group (*d* = −0.523, *p* < *0.0*01). However, significant heterogeneity was also found amongst the schizotypy, but not schizophrenia studies, as well as studies using accuracy, but not reaction time as a measure of performance. A non-significant correlation was found for the studies that examined schizophrenia symptoms or schizotypy traits as continuous variables (*r* = 0.012, *p* = 0.825). These results suggest that deficits in perceptual organization of non-fragmented stimuli are found when differences between schizophrenia/high-risk groups and comparison groups are maximized. These findings should motivate further investigation of perceptual organization abilities with closed-contour stimuli both in schizophrenia and high-risk groups, which is pertinent to current initiatives to improve the assessment and treatment of cognition in schizophrenia.

## Introduction

Schizophrenia is a severe psychotic illness, characterized by symptoms such as hallucinations and delusions, disorganized thoughts and behavior, negative symptoms (such as social anhedonia) and social/occupational dysfunction, that have a significant cost on both the person living with psychosis and society (Whiteford et al., 2013; Ettinger et al., 2014). But, despite best efforts, there are still no valid and reliable bio- or cognitive markers to aid in diagnosis or prediction of illness onset (Weickert et al., 2013). A variety of groups, including those with clinical or familial risk, have been investigated to better understand the developmental trajectory toward psychosis, given that the majority of individuals considered “at risk” do not go on to develop schizophrenia (Debbane and Barrantes-Vidal, 2014). Similarly, schizotypal personality represents a cluster of personality traits found in healthy individuals in the general community, including unusual perceptual experiences and magical thinking, odd speech or behavior and social withdrawal. These characteristics appear to be similar to, though milder than, the symptoms of schizophrenia and are considered to provide a useful index of risk for psychosis (Kwapil and Barrantes-Vidal, 2014; Mason and Claridge, 2015). Indeed, schizotypy has recently been described as an “ideal model” (Kwapil and Barrantes-Vidal, 2014) for examining the early mechanisms of risk or resistance to psychosis and can be used as an overarching framework for studying the etiology of schizophrenia (Debbane and Barrantes-Vidal, 2014; Lenzenweger, 2015). In particular, understanding the similarities and differences between high levels of schizotypy and schizophrenia may allow us to decipher the potential risk and protective factors that can lead to illness progression.

**Figure 1:**
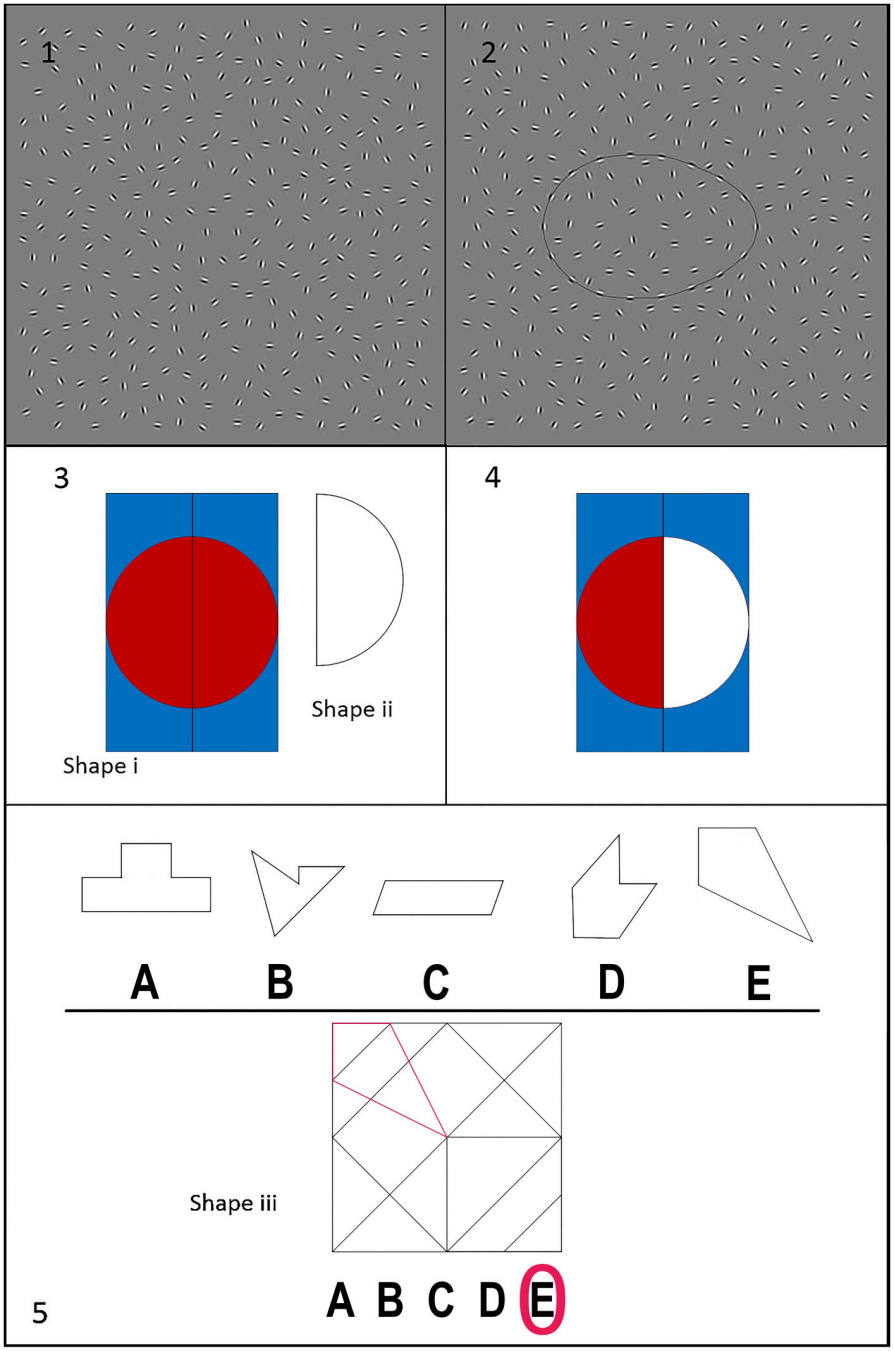
Panel **(1)/(2)** represent stimuli from the JOVI task (Personal communication from Prof. Silverstein, July 10, 2015) Panel **(1)** shows a rightward pointing “egg” shape, outlined in Panel **(2)**. Participants must correctly discriminate the direction of each fragmented stimulus. Panel **(3)** is similar to an item from the standard Embedded Figures Test (EFT; Witkin et al., 1971). The subject is required to find where “Shape ii” is inside “Shape i” by tracing it inside the shape. Panel **(4)** displays the correct answer for this item. Panel **(5)** is similar to an example from the Hidden Figures Test (HFT; Ekstrom et al., 1976), where an individual decides which of the five options **(A–E)** are found in “Shape iii” [with the correct answer **(E)** outlined on “Shape iii”]. The type of closed-contour stimulus used in Panel **(3)/(4)/(5)** is distinctly different from the fragmented stimuli shown in Panel **(1)/(2)**.

Renewed interest in the role of visual perception in schizophrenia and at-risk groups has led to a focus on impairments in *perceptual organization* (PO) as a critical domain of functioning—providing valuable insights into the underlying pathophysiology and development of the illness (Butler et al., 2008; Barch et al., 2009; Silverstein and Keane, 2011b). Perceptual organization involves “the processes by which visual information is structured into coherent patterns such as groups, contours, perceptual wholes, and object representations” (Silverstein and Keane, 2011a, p. 690). An important line of studies has examined PO in schizophrenia by measuring contour integration. The stimuli employed are fields of approximately equally spaced but randomly oriented Gabor patches, in which a target shape is represented, or sampled, by Gabor elements having orientations aligned with the contour of a target shape (Silverstein and Keane, 2011a; Silverstein et al., 2012). For the purpose of this paper we follow the terminology used by those authors where the term fragmented stimuli refers to a shape with a regularly-sampled, rather than a continuous, path. Closed-contour stimuli are those where a shape is defined by a bounding path, regardless of whether that contour is continuous or sampled. An example in the recent literature has been the development of the Jittered Orientation Visual Integration (JOVI) task^1^, which requires participants to decide whether a spatially sampled contour forms a leftward or rightward pointing shape (Figure 1). Thresholds are measured by determining the impact of added variation in sample orientation on shape-direction discrimination. This line of work has been motivated, in part, by the idea that PO impairment in schizophrenia may be most evident with fragmented, or sampled, stimuli (Uhlhaas and Silverstein, 2005). Furthermore, performance on these tasks can provide important information about the specific neural mechanisms associated with schizophrenia (Silverstein and Keane, 2011b). However, findings elsewhere in the literature suggest that PO deficits also occur with tasks involving non-fragmented (or continuous closed-contour) stimuli, (Seidman et al., 2003; Shin et al., 2006) and also the perception of intact images of faces and objects. Since PO appears to be multi-factorial in nature (Milne and Szczerbinski, 2009; Wagemans et al., 2012), focussing solely on tasks that used fragmented stimuli may inadvertently exclude other aspects of PO abilities relevant to individuals with schizophrenia and schizotypy. That is, performance on closed-contour tasks can provide valuable, complementary information about the processes involved in perceptual processing in schizophrenia and high-risk groups, such as sensitivity to changes in target shape (Almeida et al., 2014) and the contributions of borders to image segmentation (Almeida et al., 2010b). One task deserving further attention is the Embedded Figures Test (EFT; Witkin et al., 1971), which is a classic test of figure-ground segmentation based on prior Gestalt research on PO (Costa et al., 2015; Van der Hallen et al., 2015) that requires an individual to find a simple, closed-contour shape hidden within a more complex array (Witkin et al., 1971). The EFT has several advantages, including its relative brevity (typically taking 10–20 min to complete) and ease of administration in clinical settings. It draws on a number of cognitive processes, especially those related to the perception of form (as opposed to motion) and the underlying cortical mechanisms have been described (Ring et al., 1999; Manjaly et al., 2003). For example, functional imaging data shows that the local search component in the EFT involves activations of the left inferior and superior parietal cortex, as well as left ventral premotor cortex (Manjaly et al., 2003; Walter and Dassonville, 2011). Furthermore, EFT performance differs between separate clinical populations, with some (e.g., patients with schizophrenia) worse than normal and others (e.g., people with autism spectrum disorders) better than normal (Almeida et al., 2010a, Grinter et al., 2009). As a consequence it has been a popular clinical neuropsychological test for examining local processing bias. Indeed, the EFT is still widely used in research on autism (see Horlin et al., 2014, for a review), and may be particularly worthy of revisiting given the recent renewal of interest in the similarities and differences between autistic-like and schizotypal traits (Crespi and Badcock, 2008; Ford and Crewther, 2014).

In light of this, the aim of this paper was to provide a brief review and critique of the previous literature on the Embedded Figures Test (EFT) in schizophrenia, schizotypy and other at-risk groups. The EFT is traditionally described as a test of *figure-ground segregation*, which is the ability to locate an item embedded in an organized field (Witkin and Goodenough, 1976; Zelazo, 2013). For instance, participants must search for and find a simple figure, such as triangle, hidden within a larger, partially overlapping background image (see Figure 1). Individual differences in performance (reaction time and accuracy) are then described in terms of field independence (FID) and field dependence (FD). For example, individuals with slower reaction times (RT) and/or decreased accuracy are described as being “field dependent,” that is, they are thought to be more focused on the overall contextual (or “global”) material, than the spatially-localized features in the stimulus. Variations in performance can then be used to (1) make inferences about higher level visual mechanisms (Pellicano et al., 2005; Almeida et al., 2010a, 2014), (2) explore links to underlying neural mechanisms (Walter and Dassonville, 2011), and to (3) investigate how performance deficits relate to symptom presentation (Franco and Magaro, 1977) and functional outcomes (Russell-Smith et al., 2012).

The Group Embedded Figures Test (GEFT) was also developed by Witkin and colleagues, but varies from the traditional EFT in its delivery. Rather than measuring how long the participant takes to find the hidden shapes, the GEFT gives individuals a time limit to find as many hidden shapes as possible. The Hidden Figures Test (Ekstrom et al., 1976) (HFT) and Closure Flexibility test (CFT) (Thurstone and Jeffrey, 1956, 1984a,b, Hakstian and Cattell, 1976) have the same operating characteristics as the GEFT, in which participants are also required to find as many whole (closed-contour) shapes inside a complex background in a limited time. Factor analysis indicates that the GEFT and HFT load on the same factor (CFT and EFT were not tested), which suggests that these PO tasks share some underlying processes (Milne and Szczerbinski, 2009). Therefore, the scope of this review will encompass any studies using the EFT, GEFT, CFT, and HFT.

In sum, our goal was to conduct a systematic review and meta-analysis to investigate whether perceptual organization difficulties were evident for the closed-contour stimuli of the EFT, GEFT, CFT, and HFT in schizophrenia, schizotypy and other high-risk groups. This would be quantified by a significant, negative effect size across chosen studies. The meta-analysis also allowed us to explore potential between-study differences (e.g., sample characteristics) that may be masking true effects.

## Materials and methods

### Search methods

**Figure 2:**
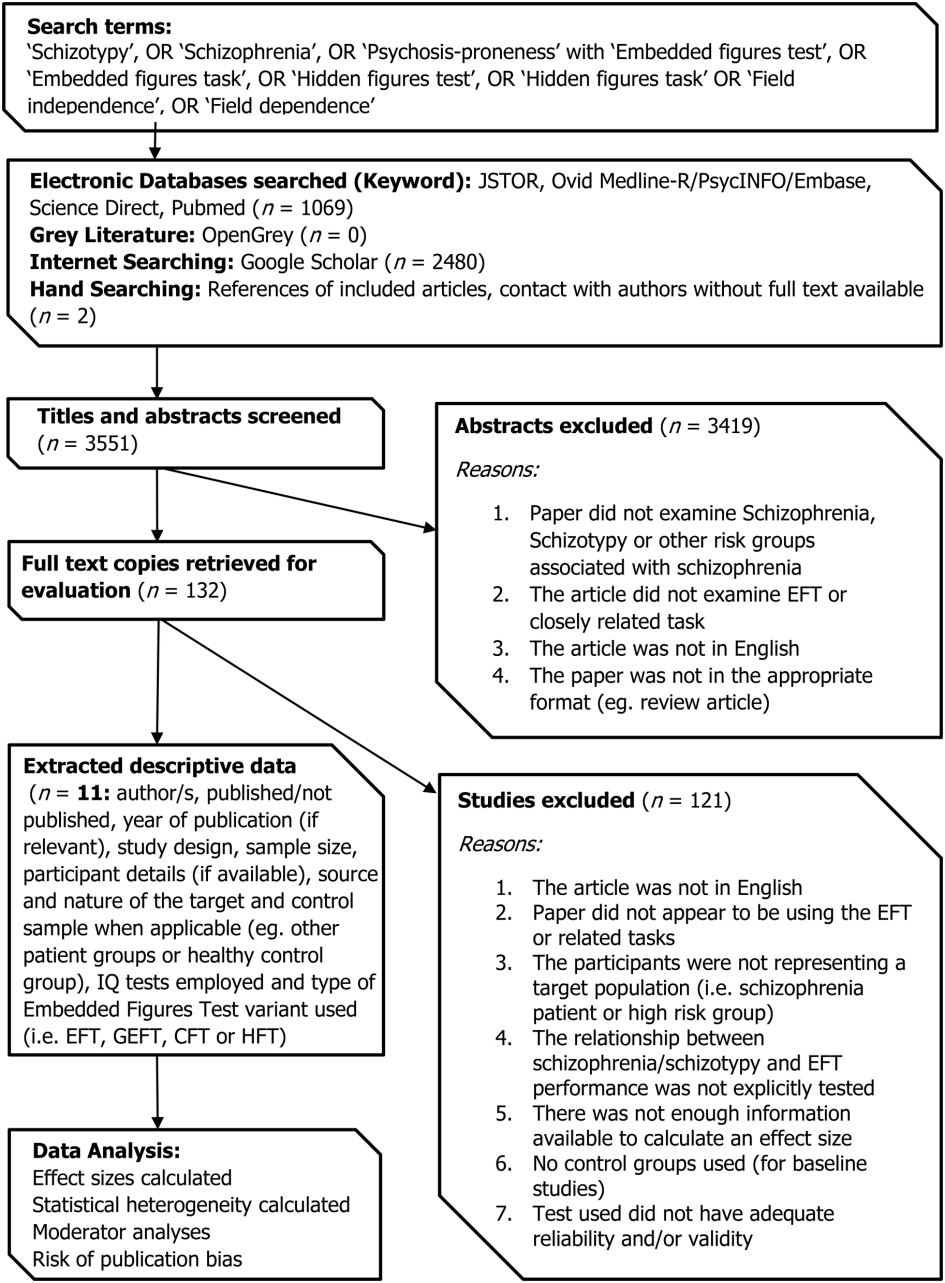
**Flow chart of search, retrieval and inclusion process**.

All data used in this meta-analysis were obtained from empirical studies, published in peer-reviewed journals. A search of the literature from 1950 to 2015 was conducted to identify relevant studies, in English. Details of the search strategy can be found in Figure 2.

### Data extraction and analysis

The data extracted and coded from the final chosen articles included: author/s, published/not published, journal and year of publication, sample size, participant details if available (age, gender, IQ), diagnosis (for patient studies), risk type (familial or psychometric), schizotypy scale measures (for psychometric high-risk studies), study design (between-groups vs. continuous), nature of the comparison sample when applicable (clinical, psychiatric, healthy) and type of test used (i.e., EFT, GEFT, CFT, or HFT).

### Study categorization

The studies included differed in design, so were split into two groups, which were analyzed separately. Specifically, one subset of studies adopted a between-group design. For example, PO ability of schizophrenia patients was compared to that of psychiatric or healthy controls or performance of high schizotypes was compared to that of low schizotypes (see Table 1A for detailed information about participant groups). The other group of studies treated symptoms of schizophrenia and schizotypy traits as continuous variables, and examined correlations between these measures with variables of interest (i.e., RT or accuracy; see Table 1B).

Table 1A**Data extracted for each study used in the meta-analysis: between-groups design**.

**Table:** 

**Author (Year)**	**Task (DV)**	**Design**	**IQ Test**	**Scale**	**Schizophrenia/high risk group**				**Comparison group**			
					**Group**	**N (%F)**	**Mean Age**	**Mean IQ**	**Group**	**N (%F)**	**Mean age**	**Mean IQ**
Bolte and Poustka, 2006	EFT (RT)	Relatives vs. controls	RSPM	–	Parents of children with EOS	36 (56)	48.8	99.7	Parents of children with ASD	62 (53)	42.6	107.2
									Parents of children with MR	30 (53)	43.5	100.6
Butler et al., 2010	CFT (#F)	Patients vs. control	QWT	SCI	Schizophrenia or schizoaffective disorder	18 (44)	38.9	98.7	Healthy	23 (48)	36.5	110.6
Cohler et al., 1977	EFT (RT)	Patients vs. controls	SILS raw score	–	Schizophrenia or schizophreniform disorder	26 (100)	31–35	29.2	Depression	14 (100)	26–30	28.6
									Healthy	44 (100)	26–30	30.7
Russell-Smith et al., 2010	EFT (RT)	High schizotypes vs. controls	WAIS	O-LIFE	High scores on unusual perceptual experiences scale	20 (75)	18.25	V: 115.6 P: 102.0	Low scores on Unusual Perceptual Experiences	20 (75)	17.7	V: 116.8 P: 110.3
									High scores on Autism Quotient	20 (75)	18.1	V: 114.4 P: 108.3
Schuldberg and London, 1989	GEFT (#C)	High schizotypes vs. controls	QWT raw score	CS	High scores on perceptual aberration-magical ideation scale (M/F split)	49 (50)	Group: 21.7	Group: 37.7	Low scores on Perceptual Aberration-Magical Ideation Scale (M/F split)	42 (50)	Group: 21.7	Group: 37.7
Schwartz, 1967	CFT (#C-#F)	Patients vs. controls	–	–	Schizophrenia	24 (–)	35.7	–	Neuropsychiatric Patients	24 (–)	37.8	–
									Healthy hospital Employees	24 (–)	37.5	–

**Table 1B:** **Data extracted for each study used in the meta-analysis: within-groups design**.

**Author (Year)**	**Task**	**Scale**	**Correlated variables**	**N (%F)**	**Mean Age**	**IQ Test**	**Mean IQ**
Braunstein-Bercovitz, 2003	CFT (#C)	SPQ	Full scale SPQ scores with CFT (#C)	58 (14)	21.3	–	–
Loas, 2004	Fr. EFT^*^ (#C)	BPRS	Positive, Negative, Disorganization, and General scale scores with Fr. EFT (#C)	62 (37)	39.7	–	–
Michalica and Hunt, 2013	CFT (#C)	O-LIFE	Cognitive Disorganization, Impulsive Non-Conformity, Introvertive Anhedonia, Unusual Experiences, and Mystical Experiences scale scores with CFT (#C)	102 (75)	19.8	–	–
Magaro et al., 1981	EFT (RT)	BPRS	Paranoid and Non-paranoid schizophrenia patients with EFT (RT)	44 (55)	18–60	SBVS	–
Tsakanikos and Reed, 2003	HFT (#C)	O-LIFE	Cognitive Disorganization, Impulsive Non-Conformity, Introvertive Anhedonia and Unusual Experiences scale scores with CFT (#C)	100 (78)	19.6	RSPM	47.94

EFT, Embedded Figures Test;

* *Fr EFT, French EFT; CFT, Closure Flexibility Test; HFT, Hidden Figures Test; GEFT, Group Embedded Figures Test. DV = Dependent Variable (RT, reaction time; #F, number of failed items; #C, number of correct items). IQ test = type of intelligence test conducted (RSPM, Ravens Standard Progressive Matrices; QWT, Quick Word Test; SILS, Shipley Institute of Living Scale; WAIS, Weschler Adult Intelligence Scale; SBVS, Stanford-Binet Vocabulary Scale). Scale = scale used to measure symptoms (in patients) or schizotypy traits (SCI, Structured Clinical Interview; O-LIFE, Oxford-Liverpool Inventory of Feelings and Experiences; CS, Chapman Scale; SPQ, Schizotypal Personality Questionnaire; BPRS, Brief Psychiatric Rating Scale). N, number of participants; “%F”, percentage of females. EOS, Early Onset Schizophrenia; ASD, Autism Spectrum Disorder; MR, Mental Retardation. Neurpsychiatric patients were composed of: anxiety reaction (n = 10), depressive reaction (n = 9), manic-depressive reaction (n = 2), psychopathic reaction (n = 1), passive-aggressive reaction (n = 1) and emotionally unstable personality (n = 1)*.

*–means data not available*.

### Statistical analyses

The Comprehensive Meta-Analysis version 2.2.064 (Borenstein et al., 2005) program was used to calculate effect sizes and to generate forest and funnel plots. A random effects model was chosen, as it accounts for differences in effect sizes that arise due to sampling demographic differences and testing variables both between and within studies (Rosenthal, 1995). Two dependent variables were recorded: reaction time and accuracy (items failed, items scored correctly). Larger reaction times, larger number of items failed and fewer items scored correctly indicate relatively worse performance. For studies using a between-groups design (i.e., schizophrenia/high-risk group vs. controls), Cohen's *d* was used to calculate between group differences. Effect sizes fall on a continuum and are considered small when *d* approaches 0.20 or less. They are considered medium when *d* = 0.50, or approach that value, large when d approaches 0.80 and very large when *d* exceeds 1.00 (Cohen, 1977). For studies exploring schizotypy traits and schizophrenia symptoms as continuous variables, Pearson's *r* was used to assess the relationship between these traits/symptoms and EFT/CFT/HFT performance. Effect sizes also continuously vary here with *r* ≤ 0.10 considered small, *r* near 0.30 considered medium and *r* ≥ 0.50 considered large (Cohen, 1977).

The heterogeneity between the selected studies was examined with Cochrane's Q, Tau^2^, and I^2^ statistics and visually through Forrest plots. A significant Q value indicates that there is a significant difference between the observed effect and the true population effect size. Since the Q can be biased by small sample size, Tau^2^, and I^2^ statistics can be used to estimate the proportion of real variance caused by confounding variables. Tau represents the standard deviation of the true effect (i.e., the variance between studies), and I^2^ indicates the percentage of the effect size that can be attributed to study differences. Possible effect size moderators were examined where significant heterogeneity was observed. The moderators selected for this analysis were: type of task used (EFT, GEFT, CFT, or HFT), dependent variable measured (reaction time or accuracy), and sample type (schizophrenia patients or high risk group).

### Risk of publication bias

Evidence suggests that significant results are more likely to be published (Egger et al., 1997), and therefore available for the meta-analysis, creating potential to bias the outcome. Publication bias was examined visually through Funnel plots to look for any asymmetries, and statistically by Eggers test of asymmetry and Rosenthal's fail-safe N. In addition, Duvall and Tweedie's *Trim and Fill* procedure was used to find the best estimate of an unbiased, overall effect size (Rosenthal, 1995).

## Results

### Description of studies

Eleven studies were extracted, all which had been published in peer-reviewed journals. The total sample size of these studies was 842, which was comprised of 257 males, 513 females, and 72 unspecified. The data from 476 participants were treated as discrete (schizophrenia/high-risk vs. healthy) and the data from 366 participants were treated as continuous variables.

**Figure 3:**
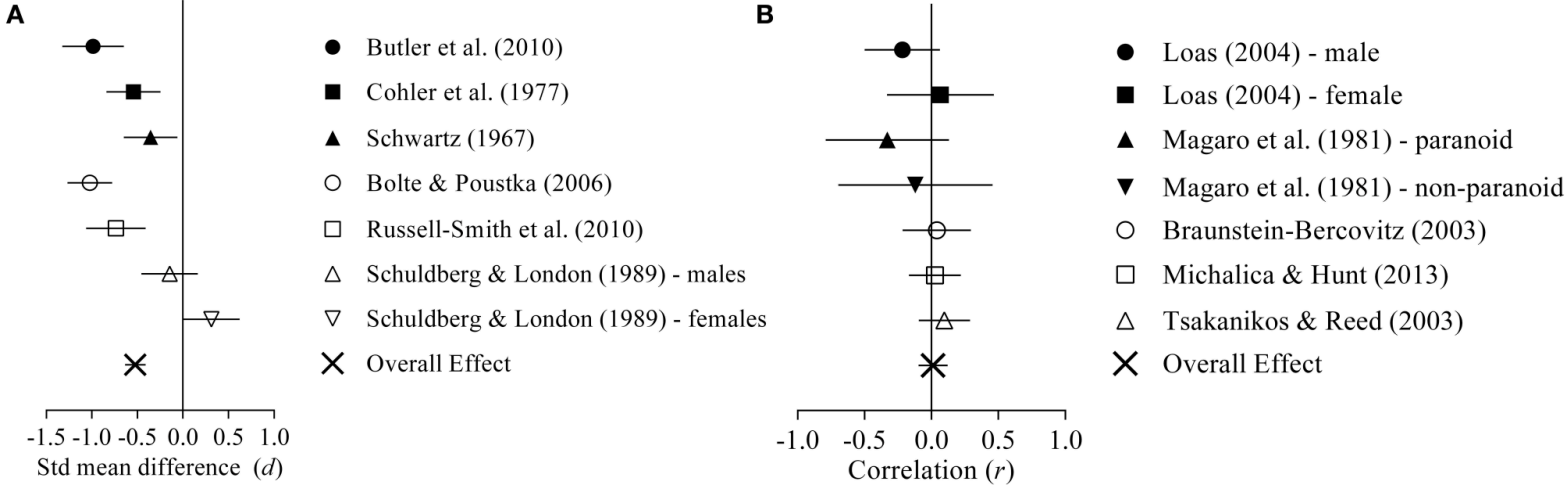
**Forrest plot of effect size and standard error for EFT/GEFT/CFT/HFT performance. (A)** Displays the studies that explored the mean difference in performance between schizophrenia (filled symbols) or high risk groups (unfilled symbols) and patient or healthy comparison groups. **(B)** Displays the studies which explored the correlation between schizophrenia symptoms (filled symbols) or schizotypy traits (unfilled symbols) with the EFT/CFT/HFT.

Overall, a significant, medium negative effect size (*d* = −0.523, *p* < 0.001; see Table 2A, Effect size statistics) was found when comparing EFT/GEFT/CFT/HFT performance between schizophrenia/high-risk groups and healthy or patient comparison groups, indicating significantly worse performance for schizophrenia/high-risk groups (see Figure 3A).

Table 2A**Mean effect size (*d*) and homogeneity statistics for EFT performance when comparing schizophrenia/high risk groups against controls**.

**Table:** 

**Domain**	**Effect size statistics**						**Homogeneity statistics**			
	***N***	***d***	**95% CI**		**Z**	***p***	**Q (df)**	***p***	**Tau**	**I^2^**
			**Lower**	**Upper**						
**Schizophrenia/High-Risk Group Vs. Controls**										
Overall effect	7	−0.523	−0.744	−0.302	−4.638	0.000	15.510 (6)	0.017	0.377	61.316
**SAMPLE TYPE -**										
High risk	4	−0.474	−0.760	−0.187	−3.243	0.001	13.181 (3)	0.004	0.544	77.241
Schizotypy	3	−0.171	−0.572	0.186	−0.937	0.349	5.403 (2)	0.067	0.411	62.984
Relatives	1	–	–	–	–	–	–	–	–	–
Schizophrenia	3	−0.596	−0.944	−0.248	−3.358	0.001	2.045 (2)	0.360	0.046	2.194
**DV MEASURE -**										
Reaction time	3	−0.805	−1.125	−0.484	−4.918	0.000	1.605 (2)	0.448	0.000	0.000
Accuracy	4	−0.268	−0.573	0.037	−1.721	0.087	8.261 (3)	0.041	0.413	63.684
**EFT VARIANT -**										
EFT	3	−0.805	−1.125	−0.484	−4.918	0.000	1.605 (2)	0.448	0.000	0.000
CFT	2	−0.626	−1.060	−0.192	−2.827	0.005	1.995 (1)	0.158	0.315	49.869
GEFT	2	0.082	−0.347	0.511	0.375	0.707	1.099 (1)	0.295	0.097	8.975

**Table 2B:** **Mean effect size (*r*) and homogeneity statistics when correlating EFT performance with schizophrenia symptoms/schizotypy traits**.

**Domain**	**Effect size statistics**						**Homogeneity statistics**			
	***N***	***r***	**95% CI**		**Z**	***p***	**Q (df)**	***p***	**Tau**	**I^2^**
			**Lower**	**Upper**						
**Continuous schizophrenia symptoms/schizotypy traits**										
Overall effect	7	0.012	−0.097	0.121	0.222	0.825	3.924 (6)	0.687	0.000	0.000

*Unable to calculate due to limited studies. All values rounded to three decimal places*.

A non-significant positive correlation (*r* = 0.012, *p* = 0.825; see Table 2B, Effect size statistics) was revealed, signaling no association between symptoms or traits and PO abilities (see Figure 3B).

### Heterogeneity

The results of examining heterogeneity between the selected studies are reported in Table 2. Cochrane's Q revealed significant heterogeneity for schizophrenia/high-risk group studies (Table 2A), but not for the correlation studies (Table 2B). For the between-group studies, an I^2^ of 61.316 was found, which indicates that 61% of the effect size could be attributed to study differences as opposed to chance. For the continuous data, an I^2^ was 0, to three decimal places of precision, which means that the effect size is unlikely to be attributed to study differences.

### Moderator analysis for between-groups studies

The dependent variable measured (accuracy) and sample type (schizotypy) exhibited significant heterogeneity, but no significant moderating effect of schizophrenia diagnosis, reaction time or task type on perceptual organization ability was observed (see Table 2A, Homogeneity Statistics).

Given the limited number of studies available, more specific comparison of different high-risk types (relatives vs. schizotypes) compared to controls must be interpreted cautiously.

### Publication bias

Egger's test was significant for the schizophrenia/high-risk group vs. control studies (intercept: 5.023; 95% CI: −10.742 to 20.789, *p* < 0.001) which indicates that there is publication bias for these studies. However, no publication bias was evident for the correlation studies (intercept:0.398; 95% CI: −2.942 to 2.146, *p* = 0.189).

Rosenthal's fail safe N indicated that a further 30 studies would be needed to create a non-significant effect for the schizophrenia/high-risk group vs. control studies. Additionally, Duvall and Tweedie's *Trim and Fill* procedure indicated that the adjusted effect sizes remained unchanged (*d* = −0.523, 95% CI: −0.301 to −0.744), thus the potential publication bias did not significantly affect results.

## Discussion

This study used meta-analytic techniques to examine evidence of deficits in segmenting closed-contour stimuli from their backgrounds in patients with schizophrenia, healthy individuals with high levels of schizotypy or other high-risk groups. A significant impairment in performance was found across the range of tasks investigated (EFT, GEFT, CFT, and HFT) compared to controls, in studies adopting a between-groups design. According to the criteria established by Cohen (1977), the magnitude of this overall deficit was moderate (*d* = −0.523, in 11 studies), and is similar in magnitude to the effect sizes reported with the JOVI task in individual studies of patients with schizophrenia (Silverstein et al., 2012, 2015)—though as yet no metaanalysis of studies using this task has been performed. By way of comparison, moderate to large effect sizes have previously been reported in meta-analyses of cognitive-perceptual functioning in people with (or at increased risk for) schizophrenia (Aleman, 2014). For instance, in schizophrenia patients, large effect sizes have been reported in studies of mismatch negativity (0.81) (Erickson et al., 2016), facial emotion perception (−0.98) (Kohler et al., 2010) and visual memory (−0.78) (Fatouros-Bergman et al., 2014). In contrast, for studies adopting a within-groups design, no evidence of an association between PO ability and schizophrenia symptoms or schizotypy scores was found. Together, these results suggest that PO difficulties are not restricted to fragmented stimuli, which has been the focus of recent task developments in the field (Silverstein et al., 2012), but are potentially constrained to studies that maximize group differences (e.g., high vs. low schizotypy).

Studies using schizophrenia and schizotypy populations were initially examined together, adhering to a dimensional model of psychosis (Nelson et al., 2013; Ettinger et al., 2014). However, as a group, these studies exhibited significant heterogeneity, prompting further analysis to identify potential sources of variability. Variability in task type (i.e., use of EFT, GEFT, CFT, or HFT) was examined, but no significant heterogeneity was found, supporting the idea that all four tasks share a common underlying substrate (Milne and Szczerbinski, 2009). Further, analysis into the measures of performance used on these tasks revealed significant heterogeneity for studies using accuracy (but not RT). The reasons underlying this outcome are not easy to explain, but likely reflect differences in methods of administration and scoring, including reporting performance in terms of number of items correct vs. items failed.

Further separating studies by sample type indicated that the largest effect of PO impairment arose in studies of patients with schizophrenia compared to controls (*d* = −0.596). A smaller (though still medium) effect size (*d* = −0.474)—with significant heterogeneity—was found in high-risk (relatives and schizotypy) samples compared to controls. However, the limited number of studies, differences in sample selection (relatives vs. schizotypes) and schizotypy measures employed (e.g., O-LIFE vs. Chapman Scales) means the data from high-risk samples must be interpreted with caution. Furthermore, unlike Schuldberg and London (1989), Bolte and Poustka (2006) and Russell-Smith et al. (2010) also endeavored to examine and/or control the influence of autism related traits on PO ability in those at increased risk for psychosis. Both studies found that individuals at increased risk for schizophrenia were slower on the EFT than those at high risk for autism. This difference in performance may feed into the recent diametric model of autism and schizophrenia, which proposes that individuals with autism or autistic-like traits have a preference for local processing, whereas individuals with schizophrenia or positive schizotypal traits have a global processing preference (e.g., Crespi and Badcock, 2008; Dinsdale et al., 2013). However, these interpretations of potentially opposing effects on PO rest on the assumption that the ability to process shapes locally or globally represent opposite ends of a single spectrum, despite the inability of the EFT to clearly separate these processes (Milne and Szczerbinski, 2009; Almeida et al., 2010a).

The current findings suggest a common impairment in performance on the EFT and related tasks, in individuals with schizophrenia and related high risk groups. Whether this represents a specific difficulty with perceptual organization of these stimuli, or reflects a more generalized deficit (e.g., due to inattention or poor motivation) in these groups cannot be determined on the basis of these findings alone. However, it is interesting to note that impairments in cognitive and perceptual processes are not inevitable in these groups (Gold et al., 2009; Chun et al., 2013; Badcock et al., 2015). For example, in a recent metaanalysis of neurocognitive performance in at-risk (schizotypal) college students, Chun and colleagues showed negligible effect sizes across a range of cognitive domains (Chun et al., 2013). This combination of results implies that it may be the case that generalized cognitive ability is relatively intact in (at least some) at-risk groups, whilst more specific functions such as those tapped by EFT and related tasks are impaired. Clearly, this possibility is still speculative and requires more detailed experimental investigation.

### Future directions

The EFT, and related tasks use closed-contour stimuli to provide a traditional measure of perceptual organization within clinical and neuropsychological literature. However, they are not measures that allow the ready assessment of individual PO processes. Rather these tasks employ combinations of different organizational cues, many of which were identified by Gestalt Psychologists in the first half of last century (Wagemans et al., 2012). Though, the EFT and related tasks are still popular in studies of autism and related disorders, they have been subject to a number of criticisms, leading to calls for improving methods of testing the critical underlying organizational processes (Almeida et al., 2010a,b, 2013, 2014). For example, Milne and Szczerbinski (2009) noted that studies using these tests tended to assume that local and global processes exist on the same continuum; thus an individual can only have a superiority on one end of the spectrum. However, following their factor analysis, Milne and Szczerbinski (2009) concluded that tasks like the EFT should be considered more narrowly as testing the ability to “disembed,” which is not directly related to global shape or motion perception (see also Almeida et al., 2010a). Similarly, atypical processing of variants of Navon compound stimuli has been demonstrated in schizophrenia and schizotypy using hierarchically organized figures, in which larger stimulus letters or numbers are composed of smaller ones (Poirel et al., 2010; Choi et al., 2014). Again, these stimuli are unable to distinguish local and global processes and, despite the use of similar terms, these stimuli seem to involve different aspects of visual organization than those assessed with the EFT (Milne and Szczerbinski, 2009). Future, studies will need to address how these different types of PO stimuli reflect the operation of underpinning perceptual processes and to what extent those processes are similar. In sum, in order to make any conclusions about *both* local and global processing, a task needed to be developed that was able to examine local and global perception separately.

Recognizing the limitations with the EFT, Almeida et al. (2010a,b, 2013, 2014) recently piloted an alternative to the EFT—the Radial Frequency Search Task (RFST)—which has been tested in a university sample exhibiting either high or low autistic-like traits. In their task, Almeida et al. employed a stimulus that can be manipulated to target either local *or* global processing. Like the EFT, the objective of the task is to find a simple shape in a complex array, with RT and accuracy used as measures of performance. Given the additional capabilities of the RFST over the EFT and the strong correlation between performance on the two tasks, future studies of PO for closed-contour stimuli in schizotypy and schizophrenia, would benefit from the inclusion of this task.

## Concluding remarks

When considered together, the evidence suggests that people with or vulnerable to schizophrenia exhibit a broad array of impairments in complex tasks where PO is likely to be central, including with both fragmented and non-fragmented stimuli. This suggests that a more targeted assessment of PO processes merits further investigation. Vision science can offer some of the most advanced tools to uncover the functional and neural mechanisms that are relevant to the perceptual anomalies found in clinical disorders (Silverstein and Keane, 2011b). With current research focused on improving methods of measuring cognition, advancing our understanding of the EFT and related tasks is an important step toward having a set of tasks that are able to measure different levels and types of perceptual deficits across the schizophrenia spectrum.

## Author contributions

KP, JB, and DB were all involved in forming the concept for this study design. KP completed the analysis and wrote the manuscript, in partial fulfillment of her PhD. JB and DB assisted with interpretation of the data and provided comments on drafts of the manuscript. KP, JB, and DB all gave final approval of the version to be published and agreed to be held accountable for all aspects of this work.

## Funding

JB is funded by the Medical Research Foundation, Perth and the Cooperative Research Centre-Mental Health. DB is supported by funding from the Australian Research Council, DP110104553.

### Conflict of interest statement

The authors declare that the research was conducted in the absence of any commercial or financial relationships that could be construed as a potential conflict of interest.

## Footnotes

^1^JOVI task can be found at http://cntracs.ucdavis.edu/task/jovi
